# Data on spatiotemporal patterns of the foundation of Japanese companies in China from 1980–2016

**DOI:** 10.1016/j.dib.2017.11.009

**Published:** 2017-11-04

**Authors:** Weiren Fan, Tomohisa Ueda, Yoshimasa Sagane

**Affiliations:** a Department of Business Science and Regional Development, Tokyo University of Agriculture, 196 Yasaka, Abashiri, Hokkaido 099-2493, Japan; b Department of Food and Cosmetic Science, Faculty of Bioindustry, Tokyo University of Agriculture, 196 Yasaka, Abashiri, Hokkaido 099-2493, Japan

**Keywords:** China, Japan, Industry, Heatmap

## Abstract

This data article provides spatiotemporal patterns of the foundation of Japanese companies in China. The data for companies in the food manufacturing, wholesaling, and service industries were collected from published lists of Chinese companies founded through the investment of Japanese companies. The data are provided in a matrix heatmap format, a two-dimensional visualization of data using color to represent the magnitude of two variables: year of foundation and area in China where the company is located.

**Specifications Table**

**Table:** 

Subject area	*Economics*
More specific subject area	*Business administration*
Type of data	*Matrix heatmap*
How data were acquired	*Processing of data sources taken from published lists of Chinese companies.*
Data format	*Analyzed*
Experimental factors	*The number of companies were accumulated from a published list.*
Experimental features	*The number of companies that are included in the matrix heatmap comprise two variables: year of foundation and area in China where the company is located.*
Data source location	*China*
Data accessibility	*Data are presented within this article.*

**Value of the data**

• The data in a heatmap format concomitantly visualize two company variables (year of foundation and area in China where the company is located), allowing researchers to capture an overview of the trends in each industry, and to compare the trends specific to each industry.
• The data can be used by researchers to examine the effects of historical events, geographical features, and Chinese policies on industrial exchanges between countries for Japan and other countries.
• The data can aid discussion on the business partnership between China and Japan.

## 1 Data

**Fig. 1:**
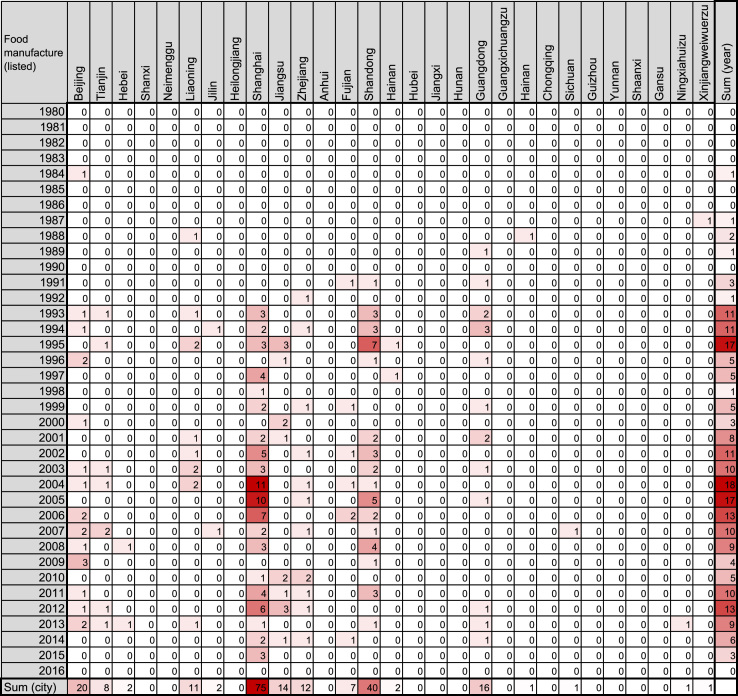
Heatmap representing the number of Chinese companies in the food-manufacturing industry founded by investment of Japanese listed companies.

**Fig. 2:**
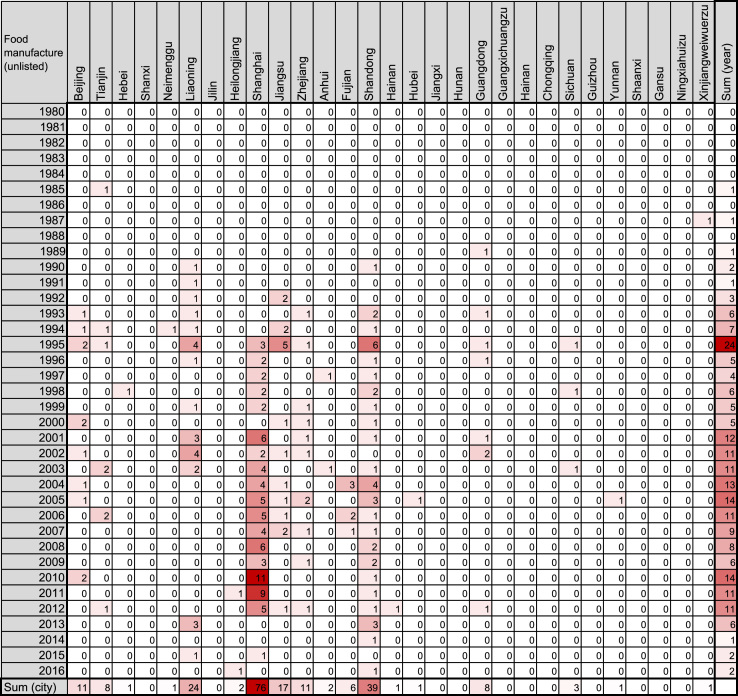
Heatmap representing the number of Chinese companies in the food-manufacturing industry founded by investment of Japanese unlisted companies.

**Fig. 3:**
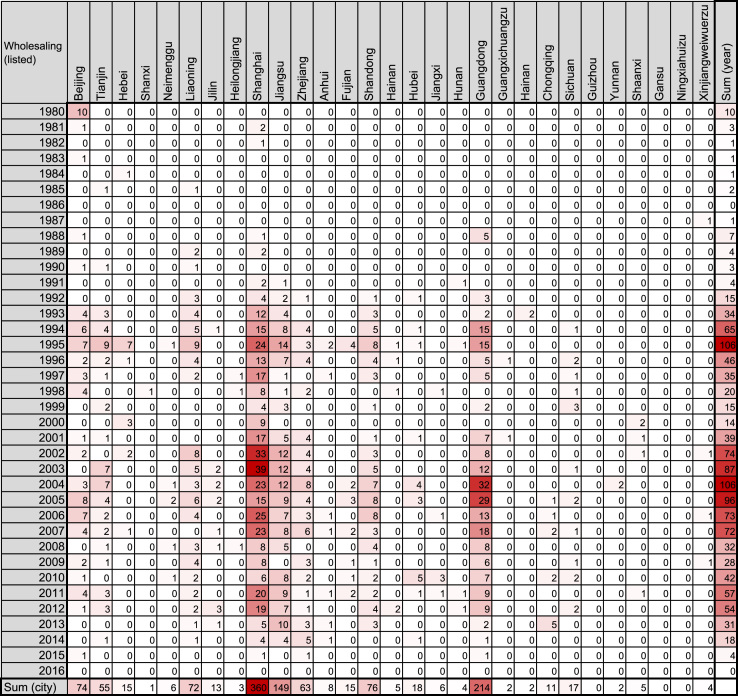
Heatmap representing the number of Chinese companies in the wholesaling industry founded by investment of Japanese listed companies.

**Fig. 4:**
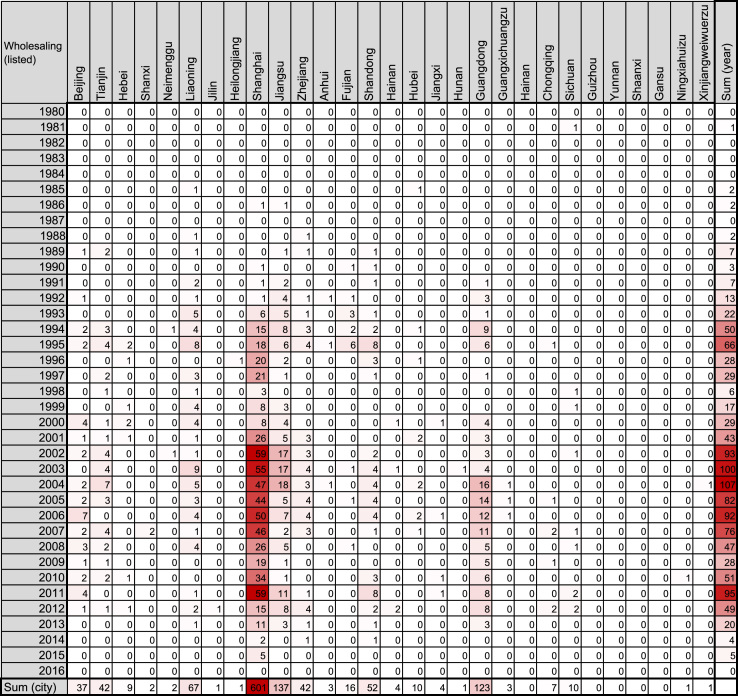
Heatmap representing the number of Chinese companies in the wholesaling industry founded by investment of Japanese unlisted companies.

**Fig. 5:**
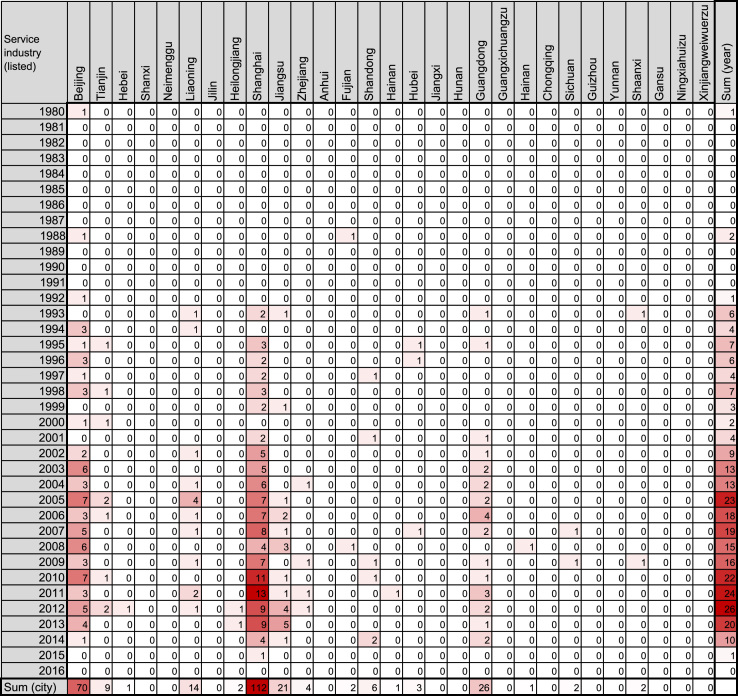
Heatmap representing the number of Chinese companies in the service industry founded by investment of Japanese listed companies.

**Fig. 6:**
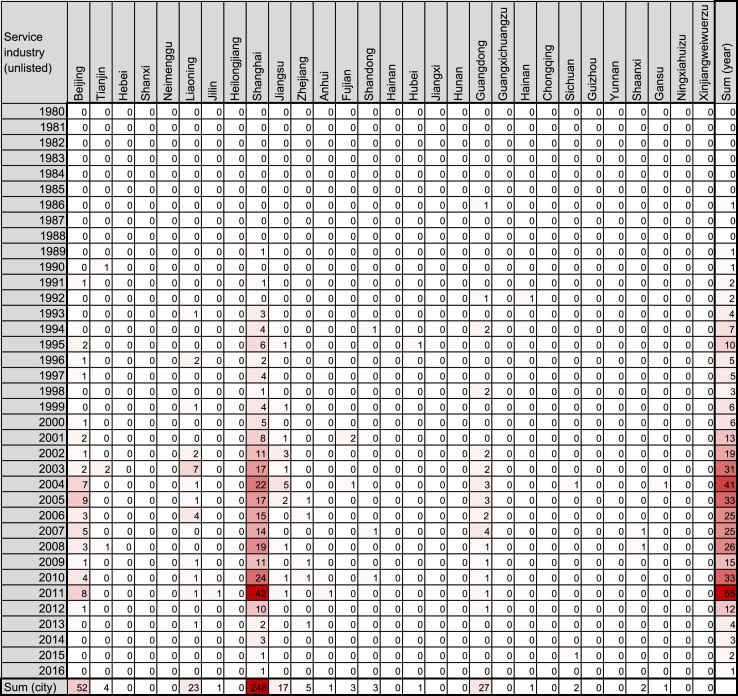
Heatmap representing the number of Chinese companies in the service industry founded by investment of Japanese unlisted companies.

To date, there have been four booms in Japanese companies’ investments in China: in 1985–1990, 1991–1999, and 2000–2007, and after 2008 [1], [2], [3]. Each boom is tightly related to factors such as historical events, geographical features, and Chinese policies. However, previous reports show line or bar graphs indicating annual amounts of investments or numbers of companies founded. The graphs do not include spatial trends, an important factor related to historical events and geographical features. Heatmaps are often employed to represent variable data, including spatiotemporal factors such as gene expression experiments [4], [5]. Hence, we employed the heatmap format (Fig. 1, Fig. 2, Fig. 3, Fig. 4, Fig. 5, Fig. 6) to visualize the spatiotemporal profiles of the foundation of Japanese companies in China. This article contains data in a heatmap format of the number of Chinese companies founded through the investment of Japanese companies. The vertical dimension indicates the year of foundation, while the horizontal dimension indicates the area in China where the companies are located. The color of the cells represents the magnitude of the number of the companies in that cell. The heatmaps for companies in the food-manufacturing industry are in Fig. 1, Fig. 2; those for wholesaling are in Fig. 3, Fig. 4. The maps for the service industry are in Fig. 5, Fig. 6. The companies were divided into two categories each, based on whether the investment company was listed (Fig. 1, Fig. 3, Fig. 5) or unlisted (Fig. 2, Fig. 4, Fig. 6).

## 2 Experimental design, materials, and methods

### 2.1 Design

According to a previous report, the food industry was included in all booms of Japanese companies’ investments in China [2]. Here, we chose the companies in the food manufacture, wholesale, and service sectors, as food industry and its related industries.

### 2.2 Source of data

The number of Chinese companies founded through investment of Japanese companies was collected from references of the 21st Century China Research Institute [6], [7] that lists such data, including established year and location, on Japanese companies investing to build Chinese companies.

### 2.3 Heatmap preparing

The number of such companies was obtained from the list in the source, and filled into a Microsoft Excel for Mac worksheet (version 15.31; Redmond, WA, USA) as shown in Fig. 1, Fig. 2, Fig. 3, Fig. 4, Fig. 5, Fig. 6. The colors of the cells were determined by the color scale option in Excel, in which the minimum number is indicated in white, and the maximum number is indicated in red. The sum of each year and area was calculated using the auto sum option in Excel. The color scale option was separately applied to a line or row of cells for sum.
